# Newly described nesting sites of the green sea turtle (*Chelonia mydas*) and the hawksbill sea turtle (*Eretmochelys imbricata*) in the central Red Sea

**DOI:** 10.7717/peerj.13408

**Published:** 2022-07-01

**Authors:** Kirsty Scott, Lyndsey K. Tanabe, Jeffrey D. Miller, Michael L. Berumen

**Affiliations:** 1 Red Sea Research Center, Division of Biological and Environmental Science and Engineering, King Abdullah University of Science and Technology, Thuwal, Makkah, Kingdom of Saudi Arabia; 2 Biological Research & Education Consultants, Unaffiliated, Missoula, MT, United States of America

**Keywords:** Sea turtles, Red Sea, Nesting beach, Marine conservation

## Abstract

**Background:**

There is relatively little published information about sea turtle nesting distribution and seasonality in the Saudi Arabian Red Sea. Upcoming large-scale developments occurring along the Saudi Arabian Red Sea coast could negatively affect many sea turtle nesting beaches with potential impacts on the survival of local populations.

**Methods:**

In 2019, two coastal beaches and three near-shore islands were surveyed for turtle nesting in the central Red Sea. We recorded all emergences, examined beach morphology, and collected sand samples to determine grain size, moisture content and colour.

**Results:**

Sea turtle nesting was found at all surveyed sites, though emergence counts were often low. The limited occurrence of nesting at several previously undocumented sites suggests that nesting activity may be widespread, but sparsely distributed, in the central Red Sea region. In addition, nesting at novel sites appeared to favour the seaward side of islands, a pattern that was not observed in previously documented areas. The substrate of most surveyed sites was composed of calcium carbonate with Ras Baridi as the only exception; it was composed of dark quartz-rich sediment. This study highlights several important sea turtle rookeries while also demonstrating that low levels of nesting occur throughout the region, although inter-annual nesting patterns still need to be determined. Future developments should be steered away from key nesting areas and the seaward bias in marginal rookeries should be taken into account where possible.

## Introduction

Nesting site distribution and beach characteristics are important to sea turtle conservation because maintaining suitable beaches is essential for the reproductive ecology of these species (Stewart, Booth & Rusli, 2019). Since there is no parental care beyond nest site selection, preferential nest placement may be the main mechanism by which sea turtles increase their fecundity and ensure the persistence of parental genes (Wood & Bjorndal, 2000; Ficetola, 2019). Various factors associated with nest placement may affect clutch success, including beach morphology and sediment characteristics like moisture content (McGehee, 1990), sediment colour (Hays, Ashworth & Barnsley, 2001), and grain size (Mortimer, 1990).

Beach morphology is important because slope and elevation prevent inundation of the nest and have energetic benefits for both the female and the hatchlings, with steeper slopes allowing females to nest closer to the sea (Horrocks & Scott, 1991). Sediment grain sizes affect the exchange of water (Chen, Wang & Cheng, 2010) between the outside environment and the egg chamber. Moisture is absorbed by the clutch during incubation and higher moisture concentration is associated with larger hatchlings (Erb, Lolavar & Wyneken, 2018), improved self-righting ability and quicker crawl speeds (Gatto & Reina, 2020). Finally, differences in sand colour can influence nest temperature with darker colours retaining more heat (Hays, Ashworth & Barnsley, 2001). Human activities that affect any of these beach characteristics could cause significant losses in both nesting and hatching success, effectively reducing the reproductive output of the regional population (Horrocks & Scott, 1991).

Five of the seven extant sea turtle species can be found in the Red Sea: the green (*Chelonia mydas*), hawksbill (*Eretmochelys imbricata*), olive ridley (*Lepidochelys olivacea*), loggerhead (*Caretta caretta*), and leatherback (*Dermochelys coriacea*) (Mancini, Elsadek & El-Alwany, 2015)*.* Of these five species, only *C. mydas* and *E. imbricata* are known to nest in the region (Mancini, Elsadek & El-Alwany, 2015)*.* While a few large nesting aggregations have been documented, specifically Ras Baridi, (Hanafy, 2012; Mancini, Elsadek & El-Alwany, 2015; Shimada et al., 2021a; Shimada et al., 2021b) and the Farasan Islands (Gladstone, 2000), sparse nesting is thought to occur along the entire Red Sea coastline (Pilcher & Al-Merghani, 2000; Miller, 2018; Shimada et al., 2021a; Shimada et al., 2021b). Potential nesting grounds at several near-shore islands and coastal sites are undescribed (Miller, 2018). Collectively, sparse nesting grounds could represent a significant reproductive input into the regional sea turtle population. However, isolation from the main breeding sites and low site attendance could leave these unstudied nesting sites vulnerable to human activities and unlikely to recover from severe disturbances.

The Saudi Arabian coastline is undergoing rapid change as part of the ‘Vision 2030’ efforts to diversify the national economy (Public Investment Fund, 2017). Large scale coastal developments (locally known as “giga-projects”) include ‘The Red Sea Project’ (a planned luxury tourist resort under construction in Al Wajh lagoon) and the futuristic city of ‘NEOM’ which is planned to encompass an area of 26,500 km^2^ in the northern Red Sea (Public Investment Fund, 2017). These developments will alter large expanses of the coastline and many nearshore islands, potentially destroying sea turtle nesting sites. While these projects aim to minimize their ecological impacts, potential damage to marginal nesting habitats will be difficult to effectively mitigate because many of these sites have not been surveyed or described.

In this study, we surveyed islands and beaches in the central Red Sea, including known sea turtle nesting grounds as well as undocumented sites with no previous records of sea turtle nesting. These surveys were used to summarise beach characteristics at each site. Beach morphology and sediment characteristics were compared between documented and undocumented nesting sites to identify differences between major and marginal rookeries. The information from this study expands our knowledge of turtle nesting distribution in the Red Sea, which is critical to mitigate the impact on sensitive species caused by the development of coastal habitats.

## Materials & Methods

### Site description

**Figure 1:**
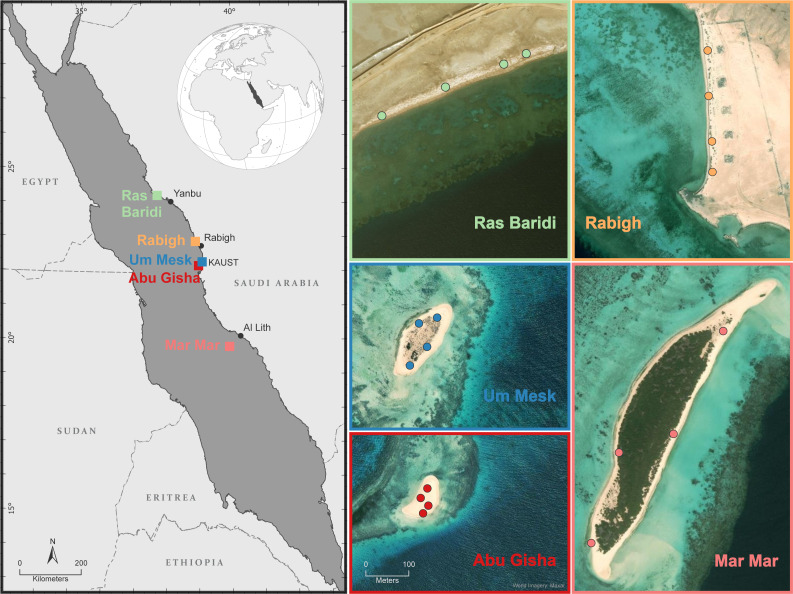
Five study sites on sandy islands and coastal beaches on the central Saudi Arabian coast of the Red Sea where turtle nesting evidence was investigated. All sites occur on the Saudi Arabian coastline on the eastern side of the Red Sea, located centrally between 24N and 22N degrees of latitude. The four small points on each map show the location of our sampling stations where we measured slope and distance to the high tide line as well as collected sand samples for moisture, grain size and colour analyses. The map bordered in light green shows Ras Baridi, the largest green turtle (Chelonia mydas) nesting beach in the Red Sea, our first documented nesting site. The map bordered in orange shows our other coastal nesting site, Rabigh. The maps bordered in blue and red show the two sandy islands located offshore of King Abdullah University of Science and Technology (KAUST), Um Mesk and Abu Gisha, respectively. Rabigh, Abu Gisha and Um Mesk are our undocumented nesting sites. Finally, the map bordered in pink is the second previously documented nesting site called Mar Mar. The scale for these five study sites can be found in the lower-left corner of the Abu Gisha map.

The study sites consist of two mainland beaches and three nearshore islands in the central Red Sea (Fig. 1). These include two known nesting grounds, one on the mainland (Ras Baridi) and one island (Mar Mar). The Ras Baridi rookery (24.273425°N, 37.626473°E) is the largest nesting site for *C. mydas* on the eastern Red Sea coast (Pilcher & Al-Merghani, 2000; Shimada et al., 2021a; Shimada et al., 2021b) and is the northernmost study site. Mar Mar (19.83576°N, 39.92635°E) is an 800-m long island in the Farasan Banks archipelago approximately 50 km from shore. It is the southernmost study site and is a known nesting ground for both *C. mydas* and *E. imbricata* (Miller, 1989). The remaining three sites, Rabigh (22.9267°N, 38.8566°E), Um Mesk (22.32464°N, 39.06938°E) and Abu Gisha (22.25303°N, 39.02822°E), have no previous records of turtle nesting, but local anecdotal data suggest that they may be undocumented rookeries (Shimada et al., 2021a; Shimada et al., 2021b). Rabigh is a 12-km long sandy peninsula with an adjacent fringing coral reef. Um Mesk is a small, vegetated island located approximately 2 km from the Saudi coastline. Abu Gisha, is also an island located approximately 4 km from shore and is nearly devoid of vegetation (Fig. 1).

### Data collection

Sampling at Um Mesk and Abu Gisha occurred every two weeks from March to November 2019 to encompass the purported nesting season of *C. mydas* and *E. imbricata* (Mancini, Elsadek & El-Alwany, 2015). The perimeter of the beaches were assessed between 09h00 and 12h00 for evidence of sea turtle nesting. Approximately 200 g of sediment were collected from the sand surface (0 cm), at *E. imbricata* nest depth (30 cm) and at *C. mydas* nest depth (50 cm) and *in-situ* measurements were taken at four sampling locations. Some sampling occasions were missed when coastguard permission could not be obtained. Between sampling occasions, sites were visited weekly to check for new tracks and nests and wooden posts were used to mark four equally spaced locations for repeated samplings along the nesting line (Fig. 1). At each study site, a nesting line was determined by evidence of body pits, or when body pits were absent the nesting line was determined by the highest point on the beach where tracks were found. Rabigh was similarly sampled every two weeks from July to November 2019. Mar Mar was sampled at the start of the nesting season during April and Ras Baridi was sampled at the end of the *C. mydas* nesting season in November 2019. These two sites were only sampled once due to permitting and having already been established as known nesting sites.

The presence of sea turtle tracks, nests, and eggshells was noted as evidence of nesting. The GPS coordinates of each location was recorded and the nesting success of each site was determined. Successful nesting attempts were classified by evidence of body pitting and camouflaging. False crawls or unsuccessful attempts were defined as tracks without evidence of body pitting or camouflaging (Silva et al., 2017). The tracks found were used to determine species by track width and gait patterns. *Eretmochelys imbricata* has an asymmetrical gait, whereas, *C. mydas* has a symmetrical gait pattern (Pritchard & Mortimer, 1999).

At Abu Gisha, the south sampling location was inundated and the sampling location was moved five metres toward the centre of the island. At each sampling location, the distance between the nesting line and the high tide line was measured and the steepest slope along the transect was measured, using a Didax clinometer. The tidal range along the central Saudi Arabian coast is relatively small (0.2 m during spring) (Herher, 2015); and was unlikely to affect data collection.

### Sediment analysis

Sand samples were subdivided into ∼100 g using a Mettler Toledo XS205 Dual Range Balance (±0.1 mg repeatability) and dried for 24 h at 65 °C in a Binder Incubator KB400 (temperature fluctuation ±0.2 °C). The sample was reweighed after drying to give percentage moisture loss content per sample. Percentage moisture loss was calculated using the formula below:

\begin{eqnarray*}\text{%}\mathrm{Moisture~ Loss}= \frac{\mathrm{wet~ weight}-\mathrm{dry~ weight}}{\mathrm{wet~ weight}} \times 100. \end{eqnarray*}

After determining the percent moisture lost, the samples were dry sieved through 8″VWR Test Sieves using a Cole-Parmer One-Touch Vibratory Sieve Shaker for three minutes at magnitude 8. The samples were sequentially sieved through four sieves with aperture widths of one mm, 500 µm, 250 µm and 125 µm and each size fraction was reweighed. Anything smaller than 125 µm fraction was discarded because of the low clay content of Red Sea sediments which would not statistically change our findings. Finally, sediment colour was determined by comparing surface sand samples to a Munsell chart (Aitkenhead et al., 2013). Munsell assignments were conducted on the same day under the same light conditions and produced numerical measures for hue, chroma, and value, allowing for quantitative comparison of sand colour among sites.

### Data analysis

All statistical analyses were conducted on R Studio Version 4.1.2 (R Development Core Team, 2022). Due to differences in the orientation of sampling locations, different tests were used to compare the coastal sites (*t*-test) and island sites (one-way analysis of variance). In both cases, the test assumptions of normality and equal variance were confirmed with Shapiro–Wilk and Levene tests. If a significant difference was found using analysis of variance (ANOVA), a Bonferroni *post hoc* correction was applied to determine the statistical significance between all pairs. When ANOVA was not possible, Bartlett’s test for homogeneity was applied to the data followed by a Kruskal–Wallis test. For all sites, a Kendall-Tau’s correlation was used to determine the correlation between slope and distance from nesting line to high tide line. Grain size and sorting were analysed using GRADISTAT Version 8.0, calculated using a log _(2)_ scale (half phi), as the sieve aperture was half of the aperture from the sieve above. Mapping was conducted using ArcGIS Pro v. 2.6.0 (Version 10.8).

## Results

### Nesting evidence

Seven nests and ten tracks were recorded at the undocumented sites between May and July 2019. Each track was regarded as one visit to the nesting site. On Abu Gisha there were two *E. imbricata* tracks and a nest, and one track where the species was undetermined. At Um Mesk there were five tracks (two *C. mydas* tracks, two *E. imbricata* tracks, and one undetermined track) and four nests (two *C. mydas* and two *E. imbricata*). Three *C. mydas* tracks were observed during the survey period at Rabigh with no evidence of successful nesting. For island sites, all individuals emerged from the western (seaward) side and all nesting events were also on the west side of the island. The nesting seasonality was partitioned by species, with *E. imbricata* nesting earlier in the season, starting in May and *C. mydas* nesting later nesting starting in late June.

### Beach morphology

The distance from the nesting line to the high tide line differed significantly between sites [Kruskal–Wallis test, H(2) = 26.695, *p* < 0.001], with Um Mesk having the largest average distance (Table 1); but also the greatest variation in nesting line/high tide line distance over the nesting season. The highest and lowest average beach slopes were found at our documented study sites. The average beach slope at Ras Baridi was 17.25° whereas the and average beach slope at Mar Mar was 7.5° (Table 1). There was a negative correlation between the angle of the beach slope and distance to the high tide line, [Kendall Tau’s correlation coefficient, *τ*b = −0.213, *p* < 0.001]. At the island sites, the slopes were generally steeper at the seaward sampling location. This trend was particularly apparent at our island sites, which typically had steeper slopes and shorter distances between nesting and high tide lines along their seaward sides.

**Table 1:** **A comparison of beach parameters measured at undocumented (Rabigh, Um Mesk, and Abu Gisha) and documented (Ras Baridi and Mar Mar) sea turtle nesting sites on the Saudi Arabian coast of the Red Sea between Mar–Nov 2019. **The range of months sampling took place during the 2019 nesting season can be found in the first row. The average slope (°), distance from the nesting line to high tide line, moisture content (%), grain size (mm), sorting (*ϕ*) calculated across the sampling period are in subsequent rows. These values are represented by the mean across the sampling period and the variation around the mean is the standard deviation. The colour match, which was evaluated with a Munsell chart, was reported in addition to the main source of the sediment at each nesting site.

	**Undocumented sites**			**Documented sites**	
	**Rabigh**	**Um Mesk**	**Abu Gisha**	**Ras Baridi**	**Mar Mar**
Months sampled (2019)	Jul–Nov	Mar–Nov	Mar–Nov	Nov	Apr
Slope (°)	14.68 ± 6.80	15.33 ± 11.77	16.88 ± 15.39	17.25 ± 16.92	7.50 ± 2.65
High tide line (m)	5.24 ± 2.07	8.62 ± 2.96	8.0 ± 4.43	7.22 ± 2.21	10.41 ± 2.45
Moisture content (%)	3.98 ± 2.18	2.75 ± 2.01	4.9 ± 3.6	3.85 ± 2.49	4.94 ± 5.41
Grain size (mm)	1.6 ± 0.18	1.43 ± 0.25	1.22 ± 0.3	1.1 ± 0.10	1.31 ± 0.30
Sorting (Φ)	0.78 ± 0.07	0.66 ± 0.08	0.67 ± 0.12	0.89 ± 0.1	0.79 ± 0.10
Colour match	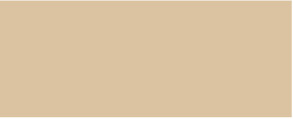	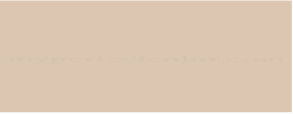	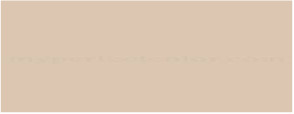	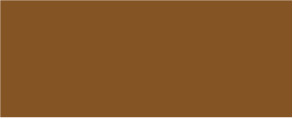	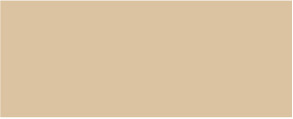
**Source**	Biogenic	Biogenic	Biogenic	Igneous	Biogenic

### Sediment characteristics

Average grain size at the different sites ranged from 1.1 mm to 1.6 mm while sorting ranged from 0.66 Φ to 0.89 Φ (Table 1). Based on these measurements, the sediment from all sampled beaches was classified as moderately sorted coarse sand. Despite the similar classification, ANOVA revealed significant differences in both grain size (*F* = 6.784, *p* = 0.0002) and sorting (*F* = 12.54, *p* < 0.01) among sites. A Bonferroni-corrected post hoc showed significance in all pairings but two and for sorting a Bonferroni-corrected post hoc was only significant for four out of all ten pairings. Ras Baridi is the most distinct site with the lowest overall grain size and overall highest sorting value.

There was not a significant difference in moisture observed between our study sites, (ANOVA, *p* = 0.988). The average moisture content was highest at Mar Mar at 4.94 ± 5.41 and lowest at Um Mesk 2.75 ± 2.01, but varied the least at Um Mesk (Table 1)*.*

Sand colour was consistent for each site throughout the study and the Munsell chart comparisons yielded similar results among most sites. The surface sediments from Um Mesk and Abu Gisha were indistinguishable and had the same colour match (Table 1, 10yr 8/2). Rabigh (10yr 8/3) and Mar Mar (10yr 8/3) samples had higher colour intensities (Table 1). Finally, Ras Baridi (10yr 4/6) was notably distinct with a larger chroma number, indicating increased colour strength, yet a lower value number, indicating a lighter overall colour (Table 1).

## Discussion

### Nesting evidence

We collected evidence of sea turtle nesting at all three novel sites described in this study, mostly from late May through early June. While the number of recorded nests was small, even limited use of these sites as reproductive habitats suggests that marginal nesting behaviour may be widespread. The Saudi Arabian portion of the Red Sea contains ∼1,150 islands (Ministry of Tourism, 2016), most of which have not been surveyed for sea turtle nesting. If a significant number of these islands are used as marginal nesting habitats, then their collective contribution to future sea turtle generations could be substantial. Additional surveys at other sites, particularly islands, could determine if marginal nesting and the seaward bias in nest selection observed at Um Mesk and Abu Gisha are widespread. New nesting sites could be related to an increase in the abundance of sea turtles over the past decade. However we must use caution because as other nesting sites worldwide exhibit fluctuations in inter-annual nesting abundances (Al-Merghani et al., 2000; Chaloupka et al., 2008). We cannot assume nesting did not occur in previous years, nor can we assess whether 2019 was a “typical” year since most sites have not been surveyed to assess temporal patterns. Future studies should include long-term nesting surveys to verify this. Confirming the existence of undocumented breeding stocks would be especially important in the Red Sea because local sea turtle populations are thought to be genetically isolated (Jensen et al., 2019) and threatened by coastal development (Mancini, Phillot & Rees, 2019; IUCN, 2020).

The human population along the coast of the Red Sea is projected to double over the next two decades (United Nations, 2017), exerting increased pressure on nesting beaches and their associated breeding areas. Establishing baselines for the widespread but sparse nesting grounds could be crucial to mitigating the worst impacts of planned developments on local sea turtle populations. Our findings highlight the shortage of information on sea turtle nesting throughout most of the Saudi Arabian Red Sea. This is not an unexpected result: Ras Baridi is the only well-studied nesting area in the region (Miller, 1997; Pilcher, 1999; Pilcher & Al-Merghani, 2000; Tanabe et al., 2020; Shimada et al., 2021a; Shimada et al., 2021b; Al-Mansi et al., 2021). Expanding research efforts at other sites should be a priority in the face of rapid coastal development. Conserving marginal sites along with large-scale rookeries while developing sustainable sea turtle ecotourism in the region will require continued scientific monitoring to assist with developing local regulations and best pratices for tour operators.

Most of our recorded nesting events occurred at the end of May and early June, which is several months earlier than the expected nesting season in the Red Sea (Shimada et al., 2021a; Shimada et al., 2021b). The effect of climate change is exacerbated in semi-enclosed basins (Lima & Wethey, 2012). Between 1982 and 2006, the sea surface temperature (SST) within the Red Sea has increased by 0.74 °C (Belkin, 2009), pushing organisms to their thermal limits (Stillman, 2003). This may also have implications for changes in sea turtle nesting seasons, because SST changes have been shown to cause shifts in nesting patterns usually resulting in earlier nesting seasons (Weishampel et al., 2003; Pike, Antworth & Stiner, 2006; Monsinjon et al., 2019). Climate change associated with the sea-level rise in the Red Sea is not predicted to be significant (Herher, 2015). However, the most vulnerable sections are the lowland plains between Yanbu and Jeddah, and the coastline around Jazan (Herher, 2015), encompassing Ras Baridi and the Farasan Islands. This is of particular concern at our low-lying study sites such as Rabigh and Ras Baridi leading to increased nest inundation that may drive sea turtles to nest in other previously unused sites.

### Beach morphology

At our undocumented nesting sites, specifically Um Mesk and Abu Gisha, the greatest angle of slope presented on the seaward side of the island with the shortest distance to the high tide line. Although the overall number of tracks and nests recorded were low, most emergences occurred on the seaward side of these islands. A steep slope and short distance to the nesting line have energetic benefits for nesting females and hatchlings (Horrocks & Scott, 1991). Furthermore, decreased time before reaching the water reduces terrestrial predation which may have benefits for hatchling production. Seaward emergences might also occur due to the turtles’ affinity to the fringing reefs on this side of the islands or merely the side closest in proximity when approaching from the open ocean. At Ras Baridi average beach slope was highest compared to other sites, this is beneficial at high-density nesting sites as steep slopes will provide elevation and may prevent nest inundation (Horrocks & Scott, 1991). Conversely, on Mar Mar there was little variation in beach slope and long crawl distances from the high tide line.

### Sediment characteristics

The sediments at all sites were composed of moderately well-sorted, coarse sand with similar moisture content (Table 1). Compared to the moisture content at sea turtle nesting sites in other regions, our moisture content was very low (McGehee, 1990), we attribute this to the small volume of annual rainfall in Saudi Arabia and small tidal range. Sediment colour and composition were also similar between most sites, with the sediments at Rabigh, Um Mesk, Abu Gisha and Mar Mar all being predominantly composed of white or light-brown calcium carbonate, most derived from the mineralizing tissues of coral reef fauna (Jerram & Petford, 2011). Um Mesk and Abu Gisha were the nearest sites to one another and the most alike, exhibiting statistically similar sediment sizes, sorting, and colouration. Mar Mar and Rabigh also had light coloured sand (Table 1), which was composed of biogenic material from nearby coral reefs (Jerram & Petford, 2011). These sites had the most similar colour matches and sorting values, surprising due to large distance between the sites.

Ras Baridi was an outlier, having the finest grain size (average = 1.1 mm) and most poorly sorted (0.89 Φ) sand of any surveyed beach. The colour at this site was noticeably darker and redder, indicating different sediment composition. Calcium carbonate was present but largely replaced by volcanic sediments, including iron-oxide rich quartz and igneous rock (Jerram & Petford, 2011). Dust from the nearby cement factory was also present, but in very small quantities, indicating that recent mitigation efforts to reduce beach contamination have been largely successful (MEPA, 1983). We hypothesise that sediment composition at Ras Baridi could be responsible for the increased sand temperature at this site compared to other Red Sea nesting sites (Tanabe et al., 2020) from increased sand albedo.

### Conservation management strategies

We recommend increased widespread surveys of offshore islands along the Saudi Arabian coast of the Red Sea to define the apparent sparse and broad-scale nesting distribution of sea turtles. Breeding sea turtles face a variety of anthropogenic threats (Lutcavage et al., 1997), so increased protection and enforcement at rookeries is needed particularly in regions where development is planned or started. Recently, Ras Baridi has been protected by Saudi Arabia’s Ba’a Foundation and a Marine Turtle Conservation Initiative has been created to protect this site. Further, the Farasan Islands has recently been designated a UNESCO Man and Biosphere Reserve. Conservation plans should be established to constantly monitor and enforce measures at this site. Based on sea turtle nesting evidence in The Red Sea Project, islands on which high-density nesting occurs will be protected and removed from development plans (Shimada et al., 2021a; Shimada et al., 2021b). Further research could include molecular studies, such as mixed stock analysis and haplotype mapping to define regional management units, and satellite tagging studies to identify migration corridors and link foraging and nesting sites (Jensen et al., 2019; Al-Mansi et al., 2021). Finally, more frequent surveys are needed to determine potential shifts in nesting seasonality, emergence success, and trends in population abundance to quantify the contribution marginal nesting sites make to the Red Sea turtle population.

## Supplemental Information

10.7717/peerj.13408/supp-1 Supplemental Information 1 Beach characteristics at sea turtle nesting sites on the Saudi Arabian Red Sea Measured beach characteristics at sandy islands and coastal beach sea turtle nesting sites, on the Saudi Arabian coast of the Red Sea during the 2019 nesting season

## Additional Information and Declarations

### Data Availability

The following information was supplied regarding data availability: The raw measurements are available in the Supplementary File.
